# *Lentinula edodes* as a Source of Bioelements Released into Artificial Digestive Juices and Potential Anti-inflammatory Material

**DOI:** 10.1007/s12011-019-01782-8

**Published:** 2019-06-29

**Authors:** Bożena Muszyńska, Katarzyna Kała, Anna Włodarczyk, Agata Krakowska, Beata Ostachowicz, Joanna Gdula-Argasińska, Piotr Suchocki

**Affiliations:** 1 Department of Pharmaceutical Botany, Faculty of Pharmacy, Jagiellonian University Medical College, Medyczna 9, 30-688 Krakow, Poland; 2 Department of Inorganic and Analytical Chemistry, Faculty of Pharmacy, Jagiellonian University Medical College, Medyczna 9, 30-688 Krakow, Poland; 3 Faculty of Physics and Applied Computer Sciences, AGH University of Science and Technology, Mickiewicza 30, 30-059 Krakow, Poland; 4 Department of Radioligands, Faculty of Pharmacy, Jagiellonian University Medical College, Medyczna 9, 30-688 Krakow, Poland; 5 Department of Bioanalysis and Drug Analysis, Faculty of Pharmacy, Medical University of Warsaw, Banacha 1, 02-097 Warszawa, Poland

**Keywords:** Shiitake, Biologically active elements, Extraction in digestive juices, Medicinal properties of mushrooms, RAW 264.7 cells

## Abstract

*Lentinula edodes* (shiitake), an edible and medicinal mushroom, was chosen for this study with the aim of evaluating the possibility of release of bioelements into artificial digestive juices and analyzing the anti-inflammatory properties. The extracts were prepared from fruiting bodies and biomass enriched with copper (Cu), zinc (Zn), and selenium (Se). The content of bioelements was analyzed by total reflection X-ray fluorescence method. Relatively low content of elements was observed in the fruiting bodies: Cu–1.6, Zn–7.6, and Se–0.12 mg/100 g d.w. compared to mycelial cultures. The anti-inflammatory properties were evaluated in RAW 264.7 cells. Based on the levels of cyclooxygenase 2 protein, nuclear factor erythroid 2-related factor 2, and peroxisome proliferator-activated receptor γ determined using Western blot technique, it was found that the addition of bioelements enhanced the anti-inflammatory properties of mycelium. This indicates that *L. edodes* cultured on a suitable medium may be used as a potential component of anti-inflammatory products.

## Introduction

*Lentinula edodes* (Berk.) Pegler (Basidiomycota), known as shiitake, is the second most popular species of edible mushroom next to *Agaricus bisporus* (J.E. Lange) Imbach (white mushroom) and is obtained by cultivation. The fruiting bodies of *L. edodes* are of both dietary and therapeutic significance, and hence, this species is listed as one of the medicinal mushrooms [1–3]. This species owes its popularity to its culinary uses, but above all to the presence of substances with therapeutic properties*.* The drugs and other medicinal products obtained from *L. edodes* are used in the treatment of various cancers due to their immunostimulating, anti-inflammatory, and anti-oxidant effects [1, 2, 4, 5]. The polysaccharides present in the fruiting bodies of *L. edodes* enhance the immune function and eliminate the side effects of chemo- and radiotherapy, besides exhibiting anti-cancer, anti-viral, and anti-bacterial properties. The compounds responsible for the biological activity of *L. edodes* are the polysaccharides belonging to the group of β-d-glucans, which include lentinan [3–9]. Other important antibiotic substances identified in the fruiting bodies of *L. edodes* are lentin (protein), lenthionine (exobiopolymer containing sulfur in the molecule), lentinosin (purine compound), and lentinamycin A and B [1]. In 1972, atherosclerotic substances, the most important of which are statins including eritadenine and lovastatin, were also detected in the fruiting bodies of *L. edodes* [6, 8, 9]. Like other mushroom species, the fruiting bodies of *L. edodes* are a good source of vitamins and have the ability to accumulate bioelements [1, 2, 5, 8]. Because elements such as copper (Cu), zinc (Zn), and selenium (Se) were detected in the fruiting bodies of *L. edodes*, which play a key role as anti-inflammatory factors, this study was undertaken to obtain biomass from the in vitro cultures of *L. edodes* with maximum content of Cu(II), Zn(II), and Se(IV) ions [2, 10–12].

The aim of this study was to investigate whether modifying the composition of the medium used for in vitro culturing of *L. edodes* will allow obtaining biomass rich in bioelements that may be used as a potential component in natural anti-inflammatory products.

The study also aimed to analyze whether the elements added to the culture media were accumulated in higher amounts by mycelium and were then released into artificial digestive juices in a Gastroel-2014 apparatus which is especially designed for this purpose [13]. It was checked whether the bioelements were released effectively under conditions imitating those prevailing in the human gastrointestinal tract, which could be reflected in their protective effects on the mucous membranes of the stomach and intestines.

The content of Cu, Zn, Se, calcium (Ca), potassium (K), iron (Fe), and manganese (Mn) was determined in mushroom materials. The content of the released bioelements was evaluated by total reflection X-ray fluorescence (TXRF) method. The study also aimed to determine which of the bioelements (Cu, Zn, Se) is the most beneficial to obtain mycelium with a higher anti-inflammatory potential, using RAW 264.7 cell lines (mouse macrophages) activated with lipopolysaccharide (LPS).

## Materials and Methods

### Reagents

The substances used to mineralization process: HNO_3_ (65%) and H_2_O_2_ (30%) (Suprapur®) were obtained from Merck (Darmstadt, Germany). Four-time distilled water with conductivity below 1 μS/cm was obtained in HLP 5 apparatus from Hydrolab (Straszyn, Poland).

Additives for mushroom media in the form of copper sulfate (CuSO_4_) and zinc sulfate (ZnSO_4_) were purchased from the Polish Company of Chemistry (Gliwice, Poland). Zinc hydrogen aspartate (C_8_H_12_N_2_O_8_Zn) and copper gluconate (C_12_H_22_CuO_14_) were purchased from Farmapol (Poznań, Poland). Selenitriglycerides (Selol)—prepared from Se(IV) and sunflower oil—was obtained from the Department of Bioanalysis and Drug Analysis at the Medical University of Warsaw in the synthesis process as described in Polish patent 76,530 [14]. Selol was used with a declared Se concentration of 5% (*w*/*v*).

The substances used to prepare artificial digestive juices were purchased from different companies: CaCl_2_ and MgCl_2_ from Chempur (Kraków, Poland); NaHCO_3_ from PPH Golpharm (Kraków, Poland); NaCl and NH_4_Cl from Alfa Aesar® (Kandel, Germany); bile salts and pepsin from BTL (Łódź, Poland); pancreatic extract and HCl Suprapur® from Merck (Darmstad, Germany); and KHCO_3_, C_6_H_8_O_7_, Na_2_HPO_4_, and K_2_HPO_4_ from Polish Company of Chemistry (Gliwice, Poland).

### Materials

Fresh fruiting bodies of *L. edodes* species of commercial origin were used for the experiments and for establishing in vitro cultures. The young fruiting bodies were taxonomically identified using the MycoKey 4.1 key (http://www.mycokey.com). Mycelial fragments were collected from the hymenial part of fresh fruiting bodies and were degreased with 70% ethyl alcohol. The fragments were then sterilized with 15% sodium hypochlorite (NaClO) solution for several minutes. After rinsing with redistilled water, sterile fragments of fruiting bodies were transferred under laminar flow to Oddoux medium solidified with agar [15]. To obtain a large amount of biomass, it is necessary that the biomass grown on in vitro cultures on solid medium be transferred to a modified Oddoux liquid substrate. Therefore, biomass obtained from in vitro cultures on solid medium was transferred to Erlenmeyer flasks containing 250 mL of liquid medium; the initial mass of the inoculum was 0.1 g. The in vitro cultures were allowed to grow for 21 days on Oddoux medium at 25 ± 2 °C, under a photoperiod of 16 h light (900 lx) and 8 h dark, on an ALTEL shaker (Poland) at 140 rpm.

### Experimental In Vitro Cultures of *L. edodes*

The following materials were prepared for the planned experiments: biomass of *L. edodes* (control) was obtained on a liquid Oddoux medium and on the same liquid medium with the addition of zinc compounds, such as zinc hydrogen aspartate (C_8_H_12_N_2_O_8_Zn) at a concentration of 200 mg/L and zinc sulfate (ZnSO_4_) at a concentration of 174.47 mg/L, and copper compounds, such as copper gluconate (C_12_H_22_CuO_14_) at a concentration of 72.88 mg/L and copper sulfate (CuSO_4_) at a concentration of 33.60 mg/L. In vitro cultures of *L. edodes* were prepared on a medium enriched with Se organic compounds too by the addition of Selol (Se(IV) 25 and 50 mg/L medium). The mycelial cultures were transferred to Erlenmeyer flasks and were shaken in order to promote a high growth of biomass. After culturing, mycelium was separated from the medium, frozen, and lyophilized (Freezone 4.5 lyophilizator, Labonco; temperature − 40 °C) for further experiments as in previous study [16, 17].

### Analysis of Bioelements in *L. edodes* Mycelium

The amount of elements in the fruiting bodies and mycelium of *L. edodes* and extracts obtained by digestion with artificial digestive juices was determined using TXRF method. The TXRF method was chosen for this study due to several significant aspects, such as high sensitivity, precision, accuracy, and repeatability of analyses, as well as possibility to achieve faster measurement with a very large number of samples. The optimization of mineralization conditions for analysis of samples in combination with the TXRF method enabled optimal analysis of bioelements in the fruiting bodies, mycelial cultures, and extracts obtained from *L. edodes*.

For this analysis, 0.2 g samples of lyophilized mushroom material were weighed, with an accuracy of 0.01 g, and were transferred to Teflon vessels, to which 2 mL of H_2_O_2_ solution (30%) and 4 mL of concentrated HNO_3_ solution (65%) were added. Mineralization was carried out in Magnum II microwave apparatus (ERTEC) in three stages of 10 min each, at a power of 70% and 100%, respectively, maintaining the temperature of the device at 290 °C. After mineralization, the solutions were transferred to quartz evaporators and evaporated on a heating plate at 150 °C to remove excess reagents and water. The residue obtained after evaporation was quantitatively transferred to 10-mL volumetric flasks with four-time distilled water. To analyze the composition of bioelements such as K, Ca, Mn, Fe, Cu, Zn, and Se in the prepared test samples, 1000 ppm gallium was used as an internal standard. The composition of elements was measured using a TXRF spectrometer Nanohunter II (Rigaku) equipped with an X-ray tube containing a molybdenum anode at 50 kV for 1000 s.

### Artificial Digestive Juice Extraction

Artificial digestive juices imitating saliva, gastric and intestinal juices were used in the study to extract the bioelements from *L. edodes* mycelium. These digestive juices were prepared as follows:

#### Artificial Saliva

Solutions of 100 mL MgCl_2_ (1.5 mmol/L), 100 mL KH_2_PO_4_ (25 mmol/L), 100 mL KHCO_3_ (150 mmol/L), 100 mL Na_2_HPO_4_ (24 mmol/L), 6 mL citric acid (25 mmol/L), and 100 mL CaCl_2_ (15 mmol/L) were prepared and added successively to a volumetric flask. The volume of the solution was made up to 1000 mL with four-time distilled water [18].

#### Artificial Gastric Juice

A total of 3.2 g of pepsin and 2.0 g of NaCl were dissolved in four-time distilled water, and then to this solution, 80 mL of 1 mol/L HCl was added. The volume of the solution was finally made up to 1000 mL with water [19].

#### Artificial Intestinal Juice

A total of 120 mg bile acids, 20 mg pancreatic extract, and 8.4 g NaHCO_3_ were dissolved in four-time distilled water, and the volume of the solution was made up to 1000 mL with water [20].

Extracts were obtained from lyophilized biomass from fruiting bodies and in vitro cultures of *L. edodes* and were digested in vitro using artificial digestive juices in the Gastroel-2014 apparatus as follows [13]. The extraction of mushroom material was carried out under conditions imitating those prevailing in the human body (temperature 37 °C and mixing movements imitating peristaltic movements). About 0.3 g of mushroom material was transferred to 100-mL Erlenmeyer flasks and moistened with 3 mL of artificial saliva solution for 1 min. Then, 20 mL of gastric juice was added to the flasks. The flasks were sealed and placed in the Gastroel-2014 apparatus for the digestion process. The process was conducted for 30 and 60 min, respectively. The obtained extracts were filtered using of paper filters and syringe membrane filters. The remaining mushroom material was again transferred to Erlenmeyer flasks, and 20 mL of intestinal juice was added. Digestion was allowed to proceed for 150 min. After digestion, the extracts were filtered to obtain a clear solution. Each sample was subjected to three independent repetitions. The content of elements was also determined by TXRF method described above.

### Preparation of Mycelial Extracts for Evaluation of Anti-inflammatory Properties

To prepare methanolic extracts of *L. edodes*, the lyophilized biomass obtained from in vitro cultures was first homogenized in a mortar, and 2 g of each homogenate sample was then used for extraction. Mycelium was extracted five times with 100 mL of methanol using ultrasound at a frequency of 49 kHz for 30 min (Sonic-2, Polsonic, Poland). Following extraction, the methanol in the extracts was evaporated to dryness. Then, 250 mg of dry extract was dissolved in 96% ethanol, filtered through sterile bacteriological syringe filters with a pore diameter of 0.2 μm, and transferred quantitatively to 5-mL volumetric flasks. The prepared samples were then diluted to the desired concentrations by mixing 100 μL with 900 μL of distilled water. Finally, the extracts were stored at 4 °C until they were used for analysis of anti-inflammatory activity in RAW 264.7 cell lines.

### Cell Cultures

*Mus musculus* macrophages RAW 264.7 (American Type Cell Culture: TIB-71) were cultured in Dulbecco’s modified Eagle’s medium supplemented with 1% antibiotic solution (100 IU/mL penicillin, 0.1 μg/mL streptomycin) and 10% fetal bovine serum (ATCC, Manassas, VA, USA). The cells were maintained in a humidified atmosphere with 5% CO_2_ in air at 37 °C and were finally seeded into a six-well plate (Sarstedt AG&Co., Nümbrecht, Germany) at a density of 5 × 10^5^ cells/well in 2 mL of medium. Before the experiment, the morphology of cells was observed with an inverted light microscope (Olympus, Tokyo, Japan), and cell viability was assessed by Trypan Blue Exclusion Test. Then, cells were activated with LPS (10 ng/mL) and incubated with the prepared mushroom extracts (100 μL) for 24 h.

No cytotoxic effects or apoptosis were observed in the RAW 264.7 cells activated with LPS and treated with *L. edodes* extracts. Cell viability varied from 100 to 99% following treatment with the extracts. After incubation, both the media and the cells were collected by scrapping.

### Western Blot for Protein Quantification

Cell lysates were prepared using M-PER (Thermo Fisher Scientific, Waltham, MA, USA) buffer with protease inhibitor cocktail set III (Merck, Darmstadt, Germany). The contents of proteins in the lysates were determined using Bradford reaction. Samples (40 μg) were solubilized in Laemmli buffer added with 2% mercaptoethanol (Bio-Rad, Hercules, CA, USA) and were then subjected to 10% SDS (sodium dodecyl sulfate)-polyacrylamide gel electrophoresis as described earlier [21]. Following transfer, membranes were blocked for 1 h at room temperature in the presence of casein in TBS (tris-buffered saline)–1% Tween buffer (Bio-Rad) and subsequently incubated overnight at 4 °C with the following primary antibodies: anti-COX-2, anti-Nrf2, anti-FABP_4_, anti-β-actin (GeneTex Inc., Irvine, CA, USA), and anti-PPARγ (Cayman Chemical, Ann Arbor, MI, USA), all of which were diluted to the ratio of 1:1000. After incubation, the membranes were washed and incubated with secondary antibodies (anti-rabbit IgG (HRP); Thermo Fisher Scientific, Waltham, MA, USA) for 1 h at room temperature. Then, the membranes were washed again, and proteins were detected using a Clarity Western ECL Luminol Substrate detection kit (Bio-Rad). The integrated optical density of the protein bands was quantified using a Chemi Doc Camera with Image Lab software (Bio-Rad).

### Statistical Analysis

The results are shown as mean ± standard deviation (SD), and all experiments were performed six times. Using one-way ANOVA with Tukey’s test was used to elaborate the results of evaluation of bioelements composition as well as proteins level. For all the tests, the value *p* < 0.05 was accepted as the level of statistical significance (GraphPad InStat).

## Results and Discussion

The amount of biomass obtained from *L. edodes* mycelium cultures grown on control media was 8–9 g d.w. (dry weight)/L medium, whereas the amount obtained from media enriched with bioelements (Cu, Se, Zn) was 9–9.5 g. The dynamics of mycelium growth did not differ from that reported in the earlier studies [16, 22].

In the first stage of the study, the content of selected macro- (K, Ca) and microelements (Mn, Fe, Cu, Zn, Se) in the fruiting bodies of *L. edodes* of commercial origin was determined, as well as those present in the mycelium of *L. edodes* obtained from in vitro cultures grown on modified liquid media, which were enriched with Cu, Zn, and Se compounds in inorganic (ZnSO_4_, CuSO_4_) and organic forms (C_8_H_12_N_2_O_8_Zn, C_12_H_22_CuO_14_, and Selol) (Table 1). It is well known that mushroom extracts exhibit anti-inflammatory properties; therefore, the bioelements that also exert similar effects were chosen for fortification of in vitro cultures [5, 22]. As a result, mycelium with the highest possible anti-inflammatory activity was obtained.

**Table 1:** Content of elements (mg/100 g d.w.) determined in the fruiting bodies and mycelium, as well as in basic media and the ones enriched with Zn, Cu and Se used for in vitro culturing of *L. edodes*

*L. edodes*	Metals							
	K	Ca	Mn	Fe	Cu	Zn	Se	Ga
*L. edodes* (fruiting bodies)	1839.5 ± 89.1a	312.2 ± 56.2a	2.9 ± 0.2a	14.5 ± 2.6a	1.6 ± 0.3a	7.6 ± 0.4a	0.01 ± 0.00	50.0 ± 0.0
*L. edodes* (control mycelium)	1035.3 ± 273.6a,b	684.4 ± 127.1a,b	18.9 ± 3.4a,b	14.5 ± 2.5b	–	17.2 ± 2.4b	0.01 ± 0.00	50.0 ± 0.0
*L. edodes* (control medium)	1828.6 ± 7.5b,c	93.4 ± 5.9a,b,c	17.5 ± 0.1a,c	12.9 ± 1.1c	–	6.0 ± 0.1b,c	–	50.0 ± 0.0
*L. edodes* + ZnSO_4_ (mycelium)	780.8 ± 51.5a,c,d	234.5 ± 26.1b,c	9.8 ± 0.8a,b,c,d	9.7 ± 1.4a,b,d	3.2 ± 0.1a,d	231.2 ± 6.5a,b,c,d	0.01 ± 0.00	50.0 ± 0.0
*L. edodes* + ZnSO_4_ (medium)	391.9 ± 17.4a,b,c,d	123.4 ± 6.9a,b,e	–	9.7 ± 1.3a,b,e	14.3 ± 0.3a,d	0.6 ± 0.3b,d,e	0.01 ± 0.00	50.0 ± 0.0
*L. edodes* + ZnHasp (mycelium)	1069.4 ± 257.4a,c,d	295.7 ± 58.6b,c,e	13.9 ± 1.3a,b,c,d	13.2 ± 3.9f	–	141.2 ± 13.8a,b,c,d,e,f	0.01 ± 0.00	50.0 ± 0.0
*L. edodes* + ZnHasp (medium)	5103.9 ± 131.6a,b,c,d	215.6 ± 55.2b,c	13.5 ± 0.2a,b,c,d	23.1 ± 0.5a,b,c,d,e,f	–	257.3 ± 0.7a,b,c,d,e,f	–	50.0 ± 0.0
*L. edodes* + CuSO_4_ (mycelium)	1002.5 ± 81.8a,c,d	362.3 ± 52.1b,c,d	15.1 ± 0.8a,b,d	11.9 ± 2.6d	21.0 ± 1.8a	11.1 ± 1.7a,b,c,d	0.5 ± 0.2a,b	50.0 ± 0.0
*L. edodes* + CuSO_4_ (medium)	2650.7 ± 132.6a,d,e	35.1 ± 5.0a,b,d,e	19.5 ± 0.1a,d,e	21.7 ± 0.6a,b,c,d,e	97.0 ± 1.0a,d,e	–	0.4 ± 0.0a,b,e	50.0 ± 0.0
*L. edodes* + CuGlu (mycelium)	1084.0 ± 103.6a,c,e,f	396.3 ± 118.8b,c	15.4 ± 0.8a,b,e	14.6 ± 4.4e,f	20.4 ± 2.3a,e,f	9.4 ± 1.5b,c,f	0.01 ± 0.0d	50.0 ± 0.0
*L. edodes* + CuGlu (medium)	391.9 ± 17.4a,b,c,d	123.4 ± 6.9a,b,d	–	9.7 ± 1.3a,b,e,f	14.3 ± 0.4a,d,e	0.6 ± 0.3a,b,c,d,f	–	50.0 ± 0.0
*L. edodes* + Se(IV) 25 mg/L medium (mycelium)	803.6 ± 16.5a,c,d	420.1 ± 39.6a,b,c	18.7 ± 1.0a,d	2.71 ± 1.5a,b,c,d	–	18.5 ± 1.7a,c,d	114.3 ± 15.3a,b,c	50.0 ± 0.0
*L. edodes +* Se(IV) 25 mg/L medium (medium)	2602.8 ± 155.2a,b,c	57.1 ± 4.4a,b,d,e	16.5 ± 0.8a	8.4 ± 0.1a,b,e,d,f	28.8 ± 0.2a,b,e	23.6 ± 1.7a,b,c,d,e	40.3 ± 3.3b,c,d,e	50.0 ± 0.0
*L. edodes* + Se(IV) 50 mg/L medium (mycelium)	621.8 ± 23.0a,b,c,e,f	359.7 ± 62.3b,c,e	14.9 ± 1.3a,b,d	13.5 ± 1.9d,e,f	7.9 ± 0.5e,f	15.1 ± 1.6a,c,d,e,f	568.6 ± 62.5a,b,c,d	50.0 ± 0.0
*L. edodes* + Se(IV) 50 mg/L medium (medium)	2599.7 ± 149.2a,b,c,d	56.18 ± 4.2a,b,d	17.3 ± 1.0.a	7.3 ± 0.8a,b,c,d,f	24.0 ± 1.2a,b,f	27.1 ± 1.3a,b,c,d,e	112 ± 15.1a,b,c,d	50.0 ± 0.0

Data are presented as mean ± standard deviation; *n* = 6 repetitions. Gallium was used as an internal standard. Tukey–Kramer test was used to reveal the differences between paired groups of elements in rows compared to fruiting bodies, control mycelium, and control medium; the same letters (a, b, c, d, e, and f) are marked for which the content differences are statistically significant (for *p* values < 0.05) (GraphPad InStat)

Among all the elements, K was found at the highest content in the fruiting bodies; its content was 1839.5 mg/100 g d.w. The content of Ca was highest in the mycelium cultured on the control medium; its content was 684.4 mg/100 g d.w. The presented results from the analysis of macroelement content in *L. edodes* mycelium were higher compared to the results obtained for fruiting bodies of the same species (K 537.9–1031.0 mg/100 g d.w., Ca 17.9–60.7 mg/100 g d.w.) [23–26]. The content of Cu was 21.0 mg/100 g d.w. in the mycelium obtained on the medium enriched with CuSO_4_, whereas the content of Fe was 14.6 mg/100 g d.w. in the mycelium obtained on the medium enriched with C_12_H_22_CuO_14_. The highest content of Mn, 18.9 mg/100 g d.w., was found in the mycelium obtained from non-enriched medium. In the case of Se, an amount of 568.6 mg/100 g d.w. was found in the mycelium obtained on the medium enriched with Selol at a higher concentration (Se(IV) 50 mg/L medium). A high amount of Zn (231.2 mg/100 g d.w.) was found in the mycelium obtained from in vitro cultures enriched with ZnSO_4_ (Table 1). Analysis of micronutrients showed that obtained *L. edodes* mycelia were characterized by a higher content of these elements than fruiting bodies of this mushroom species (Mn 0.2–4.8 mg/100 g d.w., Fe 4.4–12.5 mg/100 g d.w., Cu 1.4–18.2 mg/100 g d.w., Zn 5.9–28.4 mg/100 g d.w.) [23–26].

Because Zn, Cu, and Se, which are the elements with a high anti-inflammatory potential, were added to the culture medium, their content in mycelial cultures was analyzed in the next stage of the study, with respect to the type of compound and concentration used [5].

It was identified that the amount of Cu was highest (21.0 mg/100 g d.w.) in the biomass of *L. edodes* cultured on the medium enriched with inorganic copper salt (CuSO_4_), while a slightly lesser amount was found in the mycelium obtained from in vitro cultures grown on the media enriched with C_12_H_22_CuO_14_ (20.4 mg/100 g d.w.). A similar trend was observed for the content of Zn: the amount of Zn in the biomass of *L. edodes* obtained on the medium enriched with inorganic salt (ZnSO_4_) was 231.2 mg/100 g d.w., which was almost twice as high as the amount in the mycelium obtained from in vitro cultures grown on the medium enriched with C_8_H_12_N_2_O_8_Zn (141.2 mg/100 g d.w.). Selenium was added to the medium only in organic form due to its proven beneficial effect on the human body compared to the classic sodium selenite (Na_2_SO_3_) with a very narrow therapeutic index [12]. The highest amount of Se was obtained from the medium added with the highest dose of Se (568.6 mg/100 g d.w.). Because the elements Cu, Zn, and Se were identified in biomass obtained from in vitro cultures grown on medium enriched with these elements, a particularly valuable property of edible mushroom mycelia, i.e., their natural ability to accumulate elements from the medium on which they grow, was confirmed (Table 1) [16, 17, 26]. Therefore, mushroom mycelia can be successfully used as a natural source of these elements.

In order to determine the actual bioavailability of Cu, Zn, and Se to the human body, the release of these elements from the mushroom material into artificial digestive juices was investigated. Selenium is an essential micronutrient for human, and its deficiency causes harmful effects in living organisms, not only the amount of Zn and Cu released from the fruiting bodies and mycelium obtained from in vitro cultures grown on medium enriched with Zn and Cu salts, but also the amount of Se released from mycelium grown on medium enriched with Selol was evaluated [12]. The evaluation of the effect of addition of Se(IV) compounds on the amount of Cu and Zn released from mycelial cultures of *L. edodes* was also useful in the analysis of anti-inflammatory activity.

**Fig. 1:**
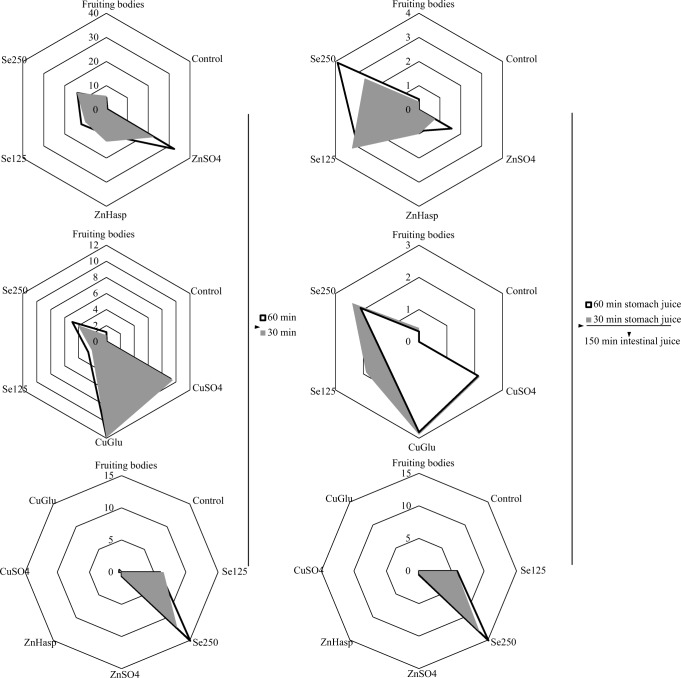
Amount (mg/100 g d.w.) of **a** Zn, **b** Cu, and **c** Se released into artificial gastric juice and **d** Zn, **e** Cu, and **f** Se released into artificial intestinal juice. Fruiting bodies—*L. edodes* fruiting bodies; Control—*L. edodes* mycelium; ZnSO_4_—*L. edodes* mycelium enriched with ZnSO_4_; ZnHasp—*L. edodes* mycelium enriched with C_8_H_12_N_2_O_8_Zn; CuSO_4_—*L. edodes* mycelium enriched with CuSO_4_; CuGlu—*L. edodes* mycelium enriched with C_12_H_22_CuO_14_; Se125—*L. edodes* mycelium enriched with Se(IV) 25 mg/L medium; Se250—*L. edodes* mycelium enriched with Se(IV) 50 mg/L medium

It was confirmed from the experiments that the tested bioelements were effectively released into artificial digestive juices. Thus, these elements may also be bioavailable for the human body (Fig. 1a–f). The previous experiments also presented that the elements from mushroom materials were released effectively [16, 17, 27].

The study showed that the examined elements were released in larger amounts into gastric juice than into intestinal juice, and to a small extent, the amount of elements released was dependent on the duration of the extraction process. The same trends were observed for the *Imleria badia* mycelium [16]. In the case of Zn, the amount released in artificial gastric juice from the biomass grown on the medium enriched with ZnSO_4_ was determined to be 22.73 mg/100 g d.w. after 30 min of incubation and 32.46 mg/100 g d.w. after 60 min of incubation. When extracted into intestinal juice, the amounts released were lower and equaled to 0.77 and 1.56 mg/100 g d.w., respectively. In the case of mycelium enriched with C_8_H_12_N_2_O_8_Zn, the amount of Zn released into gastric juice after 30 min was 13.2 mg/100 g d.w. and after 60 min was 10.3 mg/100 g d.w., and the amount released into intestinal juice was about 1 mg/100 g d.w. In the case of mycelium grown on the control medium, the amount of Zn released into artificial gastric juice was 0.4 mg/100 g d.w. In the case of Zn released into artificial intestinal juice, the amounts were found to be below the level of quantification (Fig. 1a, d). Thus, the results obtained confirmed the ability of the mycelium to accumulate Zn from the medium [16, 17]. What is important, the amounts of Zn released into the artificial gastric juice were higher than those determined in the artificial intestinal juice. This is in line with previous experiments where higher amounts of elements were released into gastric juice [27]. Some differences were observed in the results of the analysis of copper compounds. This element was more effectively released into artificial gastric juice after digestion of biomass obtained on the medium enriched with organic copper salt (C_12_H_22_CuO_14_). The content of Cu released into artificial gastric juice was determined to be 11.91 mg/100 g d.w, whereas the amount of Cu extracted into artificial intestinal juice was only 3 mg/100 g d.w. (Fig. 1b, e). In the case of Se, it was found that higher amounts (13.2 mg/100 g d.w.) were extracted into artificial gastric juice from biomass obtained from media enriched with Selol at higher concentration (Se(IV) 50 mg/L medium) than the amount (5.99 mg/100 g d.w.) obtained with Selol at lower concentration (Se(IV) 25 mg/L medium). The same tendency was observed in the analysis of Se release into artificial intestinal juice (Fig. 1c, f).

Thus, the study on the release of Cu, Zn, and Se confirmed that the fruiting bodies and mycelium of edible mushrooms are the materials from which these elements are released into artificial digestive juices and the released elements may potentially be bioavailable to the human body. The obtained results also corroborated that the biomass obtained from in vitro cultures of *L. edodes* enriched with the abovementioned microelements is a source of bioelements which ensures an average supply of these elements to meet the daily needs of the human body [28, 29].

The study also evaluated the anti-inflammatory activity of mushroom extracts obtained from the in vitro cultures of *L. edodes* grown on control media and the ones enriched with Zn, Cu, and Se compounds. The results confirmed anti-inflammatory activity of mushroom material (inflammation was induced with a commonly used agent LPS) which was in line with previous studies on edible mushroom species that demonstrated their potential anti-inflammatory effect [5, 30–34].

**Fig. 2:**
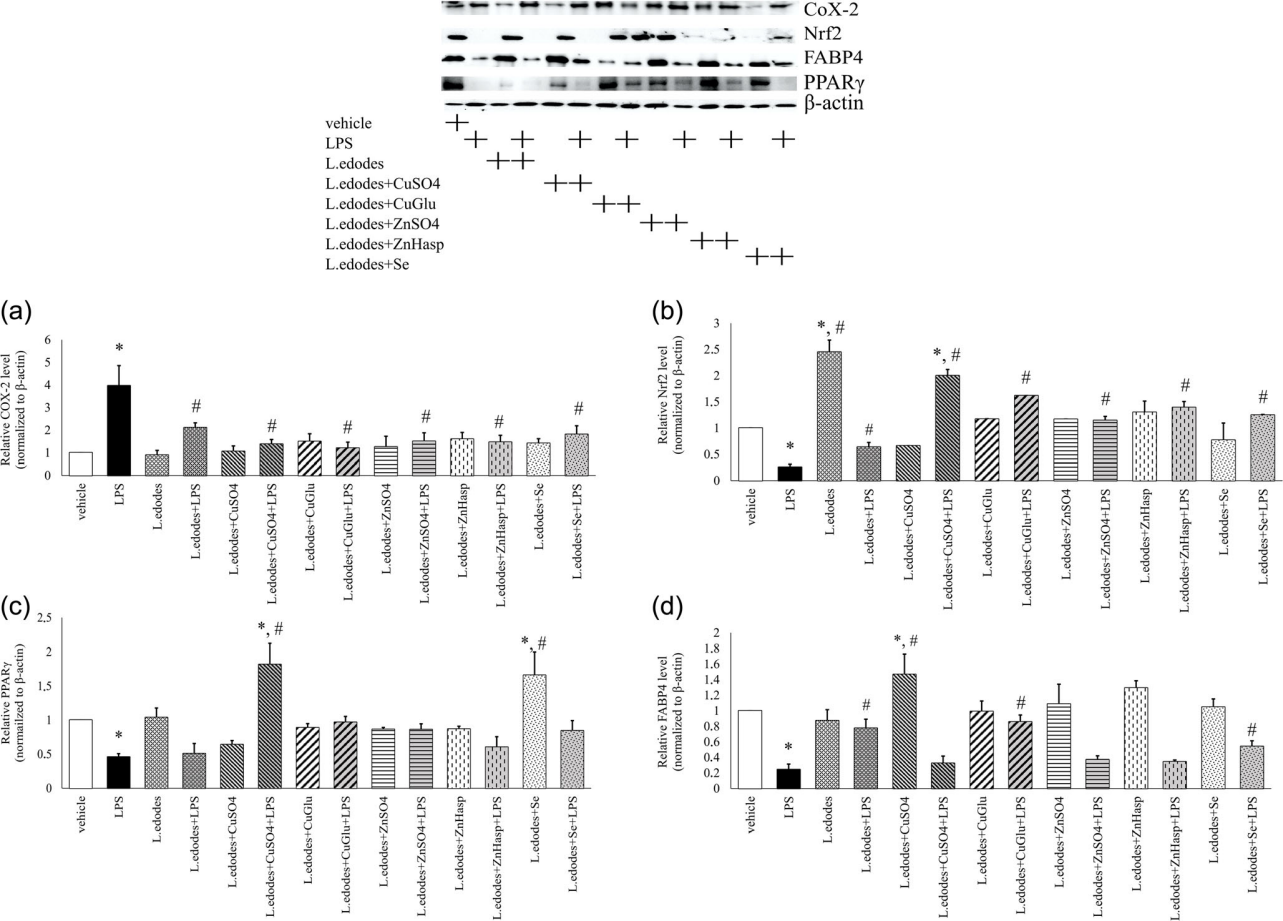
An exemplary blot and relative level of COX-2 (**a**), Nrf2 (**b**), PPARγ (**c**), and FABP4 (**d**) in RAW 264.7 cells supplemented with *L. edodes* mycelial extracts and activated with LPS. Vehicle—control cells; LPS—cells activated with lipopolysaccharide; *L. edodes*—cells incubated with extract from control mycelium; *L. edodes*+CuSO_4_—cells incubated with extract enriched with CuSO4; *L. edodes* + CuGlu—cells incubated with extract enriched with C_12_H_22_CuO_14_; *L. edodes* + ZnSO_4_—cells incubated with extract enriched with ZnSO_4_; *L. edodes* + ZnHasp—cells incubated with extract enriched with C_8_H_12_N_2_O_8_Zn; *L. edodes* + Se—cells incubated with extract enriched with Se(IV) 50 mg/L medium. Values are presented as means ± SD. Data are expressed as in relation to β-actin. *p < 0.001 relative to vehicle group, ^#^*p* < 0.001 relative to LPS-activated cells

Cyclooxygenase-2 (COX-2) is a pro-inflammatory protein, and in the light of modern knowledge, searching for natural COX-2 agonists is extremely important [32, 35–37]. The results of the present study showed statistically the highest level of COX-2 in RAW 264.7 cells activated with LPS (*p* = 0.000). A high expression of this protein was also recorded in cells incubated with control mycelium extract activated with LPS (Fig. 2a). Analysis of COX-2 level in macrophages treated with *L. edodes* extracts from in vitro cultures grown on media enriched with Cu, Zn, or Se compounds revealed that the expression of this protein was comparable to the control cells despite cells activation with LPS. Similar results were observed in the case of *I. badia* species, in which addition of zinc salt to the medium had a positive effect on the anti-inflammatory effect of mycelium compared to the control mycelium [31]. In addition, it should be emphasized that the mycelium obtained from cultures enriched with bioelements (Cu, Zn, and Se) had a protective effect on the cells, reducing their level of COX-2 in case of inflammation caused by LPS (Fig. 2a). The results observed by other researchers in *Antrodia camphorata*, *Inonotus obliquus*, *A. bisporus*, *Cantharellus cibarius*, *I. badia*, *Ganoderma lucidum*, and *Elaphomyces granulates* species showed that the signaling pathway of COX-2 was inhibited [31, 38–42]. The present study also revealed the anti-inflammatory properties of the *L. edodes* extract enriched with vitamin D in C57B1/6 mouse model with inflammatory liver disease. Supplementation of *L. edodes* extract with vitamin D resulted in a significant reduction of liver damage. In addition, the histopathological features were improved and plasma levels of inflammatory factors such as aminotransferases and interferon gamma (INF-γ) were found to decrease. It was also noted that the anti-inflammatory effect of mushroom extract and vitamin D was synergistic in nature [6].

The study also analyzed Nrf2 (nuclear factor erythroid 2-related factor 2), a protein related to detoxification processes and a transcription factor [43]. Activation of Nrf2 may lead to induction of phase II enzymes responsible for inhibiting the activity of potential carcinogens. There is also scientific evidence for the involvement of Nrf2 in extinguishing inflammatory processes [35, 36]. In addition, a direct relationship between Nrf2 activity and reduction of COX-2, which is a pro-inflammatory factor, was identified [37]. Obtaining mycelium in laboratory conditions, which can increase the activity of Nrf2, may contribute to the development of new natural drugs with anti-inflammatory potential in the future. Statistically, the highest expression of Nrf2 was found in cells incubated with control mycelial extracts (*p* = 0.000), in contrast to LPS activated cells, where each enriched mycelial extracts exhibited better anti-inflammatory activity (Fig. 2b). Especially, high expression of Nrf2 was observed in LPS-activated cells treated with mycelial extract enriched with CuSO_4_ and C_12_H_22_CuO_14_ (Fig. 2b). Comparing with the results obtained for another species, *A. bisporus*, it was found that this species also showed anti-inflammatory activity. The highest expression of Nrf2 was observed in cells treated with extracts from mycelial cultures of *A. bisporus* enriched with α-linolenic acid. This suggests that mushroom extracts enriched with α-linolenic acid may also upregulate Nrf2 signaling [44]. In addition, the results of an in vivo study conducted on *Antrodia cinnamomea* showed the positive effect of this mushroom species on the expression of Nrf2 [45]. However, the present study on *L. edodes* species showed that mainly lentinan present in this species exerted an anti-inflammatory effect, causing, inter alia, an increase in Nrf2 expression [46].

In order to confirm the anti-inflammatory effect, the expression of the transcription factor peroxisome proliferator-activated receptor γ (PPARγ) was also studied. PPARγ plays an important role in the differentiation of adipocytes, in carbohydrate and lipid metabolism, and in the regulation of inflammatory processes. It has been proven that specific PPARγ agonists can inhibit the development of insulin resistance while acting simultaneously as anti-diabetic agents, and also have beneficial effects on inflammatory diseases, and even on some cancers [47, 48]. The relationship between the presence of Zn as an anti-inflammatory agent and PPARγ activity was also demonstrated [48]. In the present study, statistically, the highest expression of PPARγ was observed in cells treated with mushroom extracts enriched with CuSO_4_ after LPS activation (*p* = 0.000) and in cells treated with extracts enriched with Selol without LPS activation (*p* = 0.01) (Fig. 2c). However, it should be emphasized that the addition of each mycelium extracts increased the level of PPARγ compared to LPS-activated control cells (Fig. 2c). A study proved that diet rich in fatty acids (FAs), especially edible mushrooms, may be a factor affecting the level of PPARγ [32]. It also proved that extracts from *C. cibarius* containing FAs exerted agonistic activity against receptors activated by PPARγ [5]. In our study, Western blot analysis of PPARγ level once again confirmed the anti-inflammatory effect of mushroom extracts.

Taking into account that edible mushrooms, including *L. edodes*, show numerous pro-health activities due to the presence of fatty acids (FAs), the expression of FA-binding protein 4 (FABP4) was studied [5]. FABP4 is mainly found in macrophages and adipose tissues, where it regulates the storage of FAs and the lipolysis process. FABP4 is also an important mediator of inflammation as well as a factor responsible for metabolism [49]. An increase the expression of FABP4 was observed in the RAW 264.7 cells treated with mushroom extract, compared to macrophages activated with LPS. The highest expression of this protein was observed in cells without inflammatory state after treatment with mushroom extracts enriched with CuSO_4_ again, while the lowest expression was observed in control cells after LPS activation (Fig. 2d). Extracts enriched in bioelements proved to be more effective than control mycelial extracts (Fig. 2d). The following are some of the saccharides in mushrooms that regulate FABP4 activity: trehalose, β-glucans (e.g. lentinan from *L. edodes* species), and chitosans [5].

## Conclusion

The experiment joining the accumulation and release of the examined bioelements into artificial digestive juices from mushroom material, and also the analysis of their anti-inflammatory properties, highlighted the potential benefits of consumption of *L. edodes* species in the form of fruiting bodies and biomass (being a potential dietary supplement). It was presented that *L. edodes* in vitro cultures accumulated Cu, Zn, and Se from modified and optimized media and released them effectively into artificial digestive juices, which can be directly translate into the expected results of digestion process in human body. This indicates the possibility and benefits of fortification of *L. edodes* mycelium with specific bioelements.

It was also demonstrated that the *L. edodes* species is characterized by anti-inflammatory properties. From the results of Western blot, it was found that addition of Cu, Zn, or Se enhanced the anti-inflammatory properties of *L. edodes* mycelial extracts, suggesting that the mycelium of *L. edodes* may be used as a potential component in natural anti-inflammatory dietary supplement. Additionally, obtained results could be used in commercial production of *L. edodes*.
